# Manual versus robotic-assisted revision total knee arthroplasty for instability: a one-year cohort analysis

**DOI:** 10.1302/2633-1462.78.BJO-2026-0018.R1

**Published:** 2026-08-05

**Authors:** Alexander Burbelo, William Stone, Trace Clark, Andrew B. Huffman, Andrew Murphy, Christopher E. Potts, Matthew Bullock, Alexander Caughran

**Affiliations:** 1 Department of Orthopaedic Surgery, Marshall University, Joan C. Edwards School of Medicine, Huntington, West Virginia, USA

**Keywords:** Revision total knee arthroplasty, Instability, Radiographic outcomes, Posterior condylar offset, Joint line restoration, Robotic-assisted surgery, revision total knee arthroplasty (TKA), revision surgery, posterior condylar offset (PCO), Joint Replacement, KOOS-JR, Total knee arthroplasty (TKA), Knee Injury and Osteoarthritis Outcome Score, primary TKA, functional outcomes, robotic-arm

## Abstract

**Aims:**

Revision total knee arthroplasty (rTKA) is a treatment option for failed primary TKA but is associated with higher complication rates and technical challenges. Robotic-assisted rTKA (rarTKA) is an emerging technique that may improve component alignment and surgical precision. However, its effect on early postoperative outcomes in rTKA for instability remains unclear.

**Methods:**

A total of 133 consecutive rTKAs for instability (82 manual and 51 robotic) performed by two fellowship-trained arthroplasty surgeons at a single institution were retrospectively analyzed. All rarTKAs used the MAKO robotic-arm system. Operating time, length of stay (LOS), joint line change, posterior condylar offset (PCO) change, and all-cause 30-/90-day complications were compared using univariable analyses. Revision implant constructs were also compared. The Knee Injury and Osteoarthritis Outcome Score for Joint Replacement (KOOS-JR) and range of motion (ROM) were assessed at three months and one year, respectively. Kaplan-Meier analysis assessed one-year reoperation-free survival.

**Results:**

RarTKA achieved a greater mean increase in PCO (3.4 mm vs 1.5 mm; p = 0.014), but required a longer operating time (186 minutes vs 173 minutes; p = 0.037). Joint line change, LOS, three-month KOOS-JR, and ROM did not differ significantly. The 30-day (4.9% vs 7.8%) and 90-day (7.3% vs 11.8%) complication rates and one-year reoperation-free survival (91.5% vs 94.1%; p = 0.900) were comparable. Although offset couplers were not used, rarTKA constructs incorporated augments more frequently.

**Conclusion:**

RarTKA demonstrated early postoperative outcomes and complication rates comparable to those of conventional manual rTKA, while achieving improved PCO restoration despite longer operating times. LOS, functional outcomes, and one-year survivorship were similar between groups. Overall, rarTKA appears to be safe and effective; however, further investigation is required to determine the long-term benefits and clinical significance.

Cite this article: *Bone Jt Open* 2026;7(8):1007–1014.

## Introduction

Total knee arthroplasty (TKA) is an effective intervention for end-stage knee osteoarthritis. However, revision TKA (rTKA) may be required for a variety of indications, including infection and instability.^1,2^ The incidence of rTKA is projected to increase by approximately 70% by 2030, and recent registry data demonstrate five-year cumulative revision rates ranging from 3% to 5%.^3,4^ While contemporary literature supports the use of robotic-assisted arthroplasty in primary TKA to improve postoperative alignment accuracy, large registry studies have not shown a corresponding reduction in revision rates compared with conventional techniques.^5,6^

In rTKA, bone loss, compromised bone quality, and ligament attenuation present significant surgical challenges.^7^ Bone loss can complicate restoration of the native joint line and impede stable implant fixation.^7^ The joint line may be difficult to accurately restore in manual rTKA due to the absence of reliable anatomical landmarks following the index procedure. Moreover, with the rise of personalized arthroplasty, the optimal reconstructive target remains debated, with competing philosophies advocating either restoration of a mechanically aligned joint line or recreation of the patient’s native alignment.^8,9^ Additionally, ligamentous laxity further complicates intraoperative decision-making, as gap balancing in flexion and extension becomes less predictable and may contribute to postoperative instability.^7^

Robotic-assisted rTKA (rarTKA) represents a promising advancement in arthroplasty, offering potential benefits over conventional techniques such as objective assessment of flexion and extension gaps throughout range of motion (ROM) and enhanced understanding of 3D component positioning and its impact on overall knee balance.^10-12^ While robotic assistance has been extensively studied in primary TKA, demonstrating improved joint line restoration, enhanced femoral component positioning, and reduced average hospital length of stay (LOS),^13,14^ its application in rTKA remains largely uncharted. RarTKA may be particularly valuable in cases of instability, which accounts for up to 14% of revisions,^15,16^ as implant malposition and loss of knee balance are associated with earlier revision rates and decreased patient satisfaction.^9,10,17^ These technical challenges underscore the need for improved precision and reproducibility in component placement, driving interest in robotic assistance for complex revision cases.

The objective of this study was to compare perioperative, radiological, and short-term postoperative outcomes between rarTKA and conventional manual rTKA performed for instability. The primary outcomes were radiological measures of reconstruction, including joint line deviation and posterior condylar offset (PCO). Secondary outcomes included operating time, LOS, 30- and 90-day postoperative complications, patient-reported outcomes (Knee Injury and Osteoarthritis Outcome Score for Joint Replacement (KOOS-JR)),^18^ ROM, and one-year reoperation-free survivorship. We hypothesized that patients undergoing rarTKA would demonstrate balanced gaps and achieve outcomes comparable to those of manual rTKA with respect to hospital LOS and readmission rates, with no significant differences in one-year reoperation-free survivorship. Additionally, we sought to describe differences in revision implant constructs between the two groups.

## Methods

### Patient cohort

This retrospective review included 133 patients (82 manual rTKA and 51 rarTKA), identified from the local electronic medical record system. Inclusion criteria consisted of patients who underwent rTKA for instability between July 2018 and December 2025. Patients undergoing rTKA for infection, conversion of unicompartmental knee arthroplasty to TKA, exchange of only the polyethylene or patellar components, or periprosthetic fracture were excluded. All procedures were performed by two fellowship-trained surgeons (AC, MB) at a single institution. The mean follow-up for the study population was 2.24 years (SD 1.78); however, outcomes for both groups were evaluated up to one year postoperatively. Institutional Review Board approval was obtained for this study. Given the retrospective nature of the study and use of de-identified data, the requirement for informed consent was waived by the Institutional Review Board. This study was conducted and reported in accordance with Strengthening the Reporting of Observational Studies in Epidemiology (STROBE) guidelines for observational studies.^19^

Patients were selected to undergo rarTKA or manual rTKA based on surgeon preference, case availability, and the temporal adoption of robotic technology at our institution. No standardized criteria were used to assign patients to either treatment group.

### Surgical technique

Manual rTKA procedures were performed using conventional revision instrumentation and intramedullary femoral and tibial cutting guides, in accordance with the manufacturer’s recommended surgical technique. Gap balancing during manual rTKA was typically achieved through preoperative templating and intraoperative surgeon judgement using conventional alignment techniques. Adjunctive components, such as stems, offsets, augments, metaphyseal cones, and constrained polyethylene components, were employed as indicated to optimize implant fixation and knee stability (Table I).

**Table I.** Summary of revision constructs.

Variable	Manual (n = 82)	Robotic (n = 51)	p-value*
**Polyethylene liner, n (%)**			< 0.001
Posterior stabilized	11 (13.4)	19 (37.3)	
Constrained	71 (86.6)	32 (62.7)	
Median polyethylene size, mm (IQR)	13 (9 to 18)	11 (9 to 16)	0.095
**Femoral stem length, n (%)**			< 0.001
Short (50 mm)	39 (50.0)	6 (15.8)	
Long (≥ 100 mm)	39 (50.0)	32 (84.2)	
**Tibial stem length, n (%)**			0.630
Short (50 mm)	54 (69.2)	31 (64.6)	
Long (≥ 100 mm)	24 (30.8)	17 (35.4)	
Femoral offset used	24 (29.3)	0 (0)	< 0.001
Tibial offset used	8 (9.6)	0 (0.0)	0.020
Distal femoral augments	60 (73.2)	50 (98.0)	< 0.001
Posterior femoral augments	29 (35.4)	44 (86.3)	< 0.001
Tibial augments	40 (48.8)	32 (62.7)	0.140
Femoral cone used	24 (29.3)	2 (3.9)	< 0.001
Tibial cone used	70 (85.4)	37 (72.5)	0.090

* Categorical variables were compared using Fisher’s exact test and continuous variables using the Mann-Whitney U test. A p-value < 0.05 was considered statistically significant.

N/A, not applicable.

The rarTKA surgical technique used in this study was previously described in a published case series.^20,21^ Preoperatively, a CT scan was obtained and formatted according to manufacturer protocol. During surgery, femoral and tibial tracking arrays were placed strategically, with the tibial array positioned extraincisionally and the femoral array placed intra-incisionally within the distal femoral metaphysis to avoid interference with implant removal or insertion. Next, the mechanical axis of the operating limb was established using anatomical landmarks, including the medial and lateral malleoli and centre of the femoral head.

Following removal of the existing polyethylene insert, registration was performed using 40 points at the femoral implant and distal femur and 40 points of the tibial component and proximal tibia to align with the preoperative CT model. With the original polyethylene component secured to the tibial baseplate, medial and lateral flexion and extension gaps were assessed near full extension and at approximately 90° of flexion. Virtual revision implants were adjusted on the planning screens to achieve symmetrical flexion and extension gaps. After careful removal of the existing implants, bony resections were performed using the MAKO robotic-arm system (Stryker, Michigan), including any necessary cuts for augmentation. In both groups, prior to trialling, manual instrumentation was used for metaphyseal cone preparation when necessary. Cementless cones were employed for metaphyseal defects, and all revision implants were fully cemented.

### Perioperative data collection

Patient demographic details and baseline characteristics were collected, including age, sex, BMI, and American Society of Anesthesiologists (ASA) grade.^22^ Surgical variables, such as technique (manual vs robotic) and indication for revision (mechanical complication or instability) were confirmed from operative reports. Intraoperative variables included anaesthesia type (general vs regional), procedure duration, and any associated complications. Early postoperative outcomes included hospital LOS and all-cause 30- and 90-day complication rates, defined as any emergency department visit or hospital readmission following revision surgery (Table II).

**Table II.** Baseline characteristics and perioperative outcomes.

Variable	Overall (n = 133)	Manual (n = 82)	Robotic (n = 51)	p-value*
Mean age, yrs (SD)	65.9 (8.6)	66.4 (9.0)	65.0 (7.9)	0.363
**Sex, n (%)**				0.890
Female	70 (52.6)	43 (52.4)	27 (52.9)	
Male	63 (47.4)	39 (47.6)	24 (47.1)	
**ASA grade, n (%)**				0.840
II	22 (16.5)	13 (15.9)	9 (17.6)	
III to IV	111 (83.5)	69 (84.1)	42 (82.4)	
Regional anaesthesia, n (%)	92 (69.2)	55 (67.1)	37 (72.5)	0.450
Mean BMI, kg/m² (SD)	34.4 (7.3)	34.4 (7.2)	34.3 (7.5)	0.940
Mean preoperative ROM, ° (SD)	106.6 (16.6)	108.5 (11.9)	103.0 (23.1)	0.126
Mean preoperative KOOS-JR (SD)	48.2 (13.2)	49.6 (13.4)	47.4 (13.3)	0.686
Mean operating time, mins (SD)	178 (SD 33)	173 (SD 35)	186 (SD 27)	0.037
**Length of stay, days, n (%)**				0.680
1	113 (85.0)	72 (87.8)	41 (80.4)	
>1	20 (15.0)	10 (12.2)	10 (19.6)	
Mean 2 week ROM, ° (SD)	95.8 (19.9)	97.5 (19.5)	92.3 (20.6)	0.267
Mean 3 month KOOS-JR (SD)	60.9 (17.3)	60.6 (19.8)	61.3 (12.6)	0.915
Mean joint line deviation, mm (SD)	1.0 (4.9)	1.3 (4.8)	0.6 (5.0)	0.451
Mean posterior condylar offset change, mm (SD)	2.3 (3.8)	1.7 (3.8)	3.4 (3.6)	0.014
**Complications, n (%)**				
30 days	8 (6.0)	4 (4.9)	4 (7.8)	> 0.900
90 days	12 (9.0)	6 (7.3)	6 (11.8)	> 0.900

* Compared between the manual and robotic groups using Welch two-sample *t*-test. Categorical variables expressed as proportions, compared using Fisher’s exact test. A p-value < 0.05 was considered statistically significant.

ASA, American Society of Anesthesiologists ; KOOS-JR, Knee Injury and Osteoarthritis Outcome Score for Joint Replacement; ROM, range of motion.

Patient-reported functional outcomes included KOOS-JR, assessed preoperatively and at three months postoperatively. Knee ROM was compared across preoperative measurements, two-week postoperative values, and one-year follow-up. ROM was assessed in the clinical setting using a goniometer by the treating surgeon at each postoperative timepoint.

Rates of rerevision and manipulation under anaesthesia (MUA) were assessed at one year and used to calculate 365-day reoperation-free survival with a log-rank test.

**Fig. 1:**
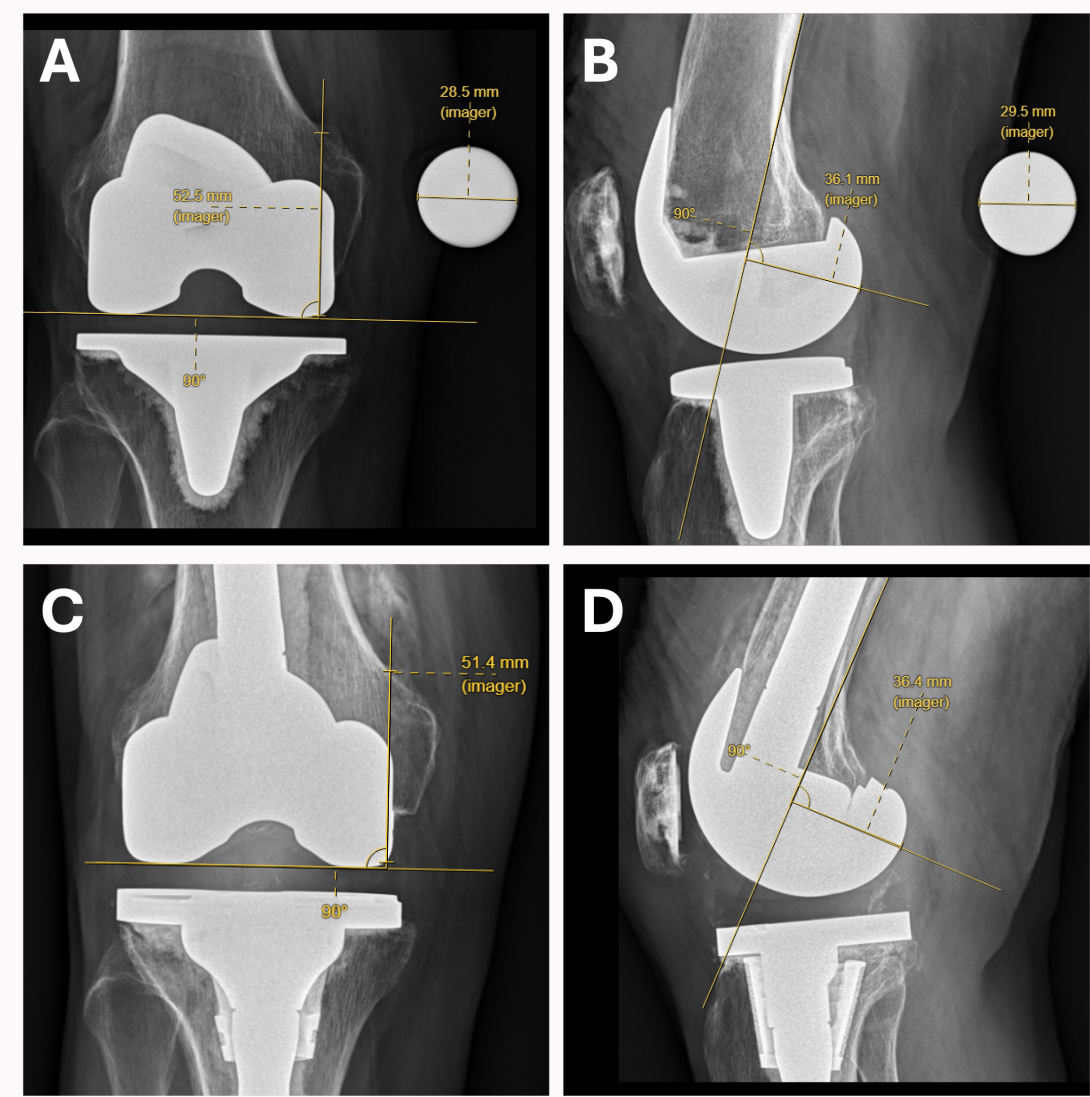
Anteroposterior (AP) and lateral radiographs of the total knee arthroplasty of a 64-year-old male at (a and b) prerevision, and (c and d) six weeks post-revision. (a and c) AP radiographs were used to calculate joint line position, while (b and d) lateral radiographs were used to measure posterior condylar offset at the corresponding timepoints.

Anteroposterior and lateral radiographs obtained prerevision (Figures 1a and 1b) and six weeks post-revision (Figures 1c and 1d) were used to calculate changes in joint line position and PCO for patients in each group. The joint line was measured relative to the adductor tubercle on the anteroposterior radiographs, while PCO was assessed on the lateral radiographs using previously described methods.^23,24^ All radiological measurements were performed by a study investigator (TC) who was not involved in the index surgical procedure. The investigator was blinded to clinical outcomes at the time of measurement.

### Statistical analysis

Continuous variables were reported as means with SD, and categorical variables were presented with corresponding proportions. All statistical analyses were conducted using R v. 4.4.3 (RStudio, USA). Continuous variables presented as means were compared between the manual and robotic groups using Welch two-sample *t*-test, whereas continuous variables presented as medians were compared using the Mann-Whitney U test. Categorical variables were compared using Fisher’s exact test. All tests were two-tailed. A p-value < 0.05 was considered statistically significant.

## Results

The cohort had a mean age of 65.9 years at time of surgery with a mean BMI of 34.4 kg/m^2^ (Table II). In all, 53% of patients were female, and 83.5% had an ASA grade of III or IV (Table II). There were no significant differences between groups with respect to age, sex, BMI, ASA grade, preoperative KOOS-JR scores, or anaesthesia type (regional vs general).

Operating time differed significantly between groups, with the manual cohort averaging 173 minutes and the robotic-assisted cohort averaging 186 minutes (p = 0.037; Table II). Both groups had a similar proportion of patients with a LOS > one day (12.2% vs 19.6%; p = 0.680). The mean joint line change was comparable between groups, measuring 1.3 mm (SD 4.8 in the manual cohort and 0.6 mm (5.0) in the robotic-assisted cohort (p = 0.451) (Table II). Patients undergoing rarTKA had a significantly greater mean increase in PCO compared with the manual group (3.4 mm vs 1.7 mm; p = 0.014).

Both cohorts primarily used constrained polyethylene inserts rather than posterior-stabilized designs. The mean insert thickness was 11 mm in the robotic group compared with 13 mm in the manual group (p = 0.095; Table I). The robotic group used significantly longer femoral stems (p < 0.001), whereas tibial stem length was comparable between groups (p = 0.630). Notably, offset couplers were not used in the robotic group; in contrast, 30% of manual cases used femoral offset (p < 0.001), and nearly 10% used tibial offset (p = 0.020). Overall, the robotic cohort used more augments, including distal femoral (p < 0.001), posterior femoral (p < 0.001), and tibial augments (p = 0.140), and fewer femoral metaphyseal cones (p < 0.001) compared with the manual group.

Postoperative complications were comparable between groups, with 4.9% and 7.8% of patients experiencing a complication within 30 days (p > 0.900) and 7.3% and 11.8% within 90 days (p > 0.900) for the manual and robotic cohorts, respectively (Table II). When stratified by Clavien-Dindo classification, there were no differences in complication severity between groups (p = 1.000).

Within the first 30 days, manual group complications included joint infection, dyspnea, cellulitis, nausea, and gastrointestinal issues, with one patient requiring admission for revision due to infection. In the robotic group, complications were limited to chest pain and gastrointestinal issues, with no admissions. Between 30 and 90 days, manual group complications included vertigo, cellulitis, nephrolithiasis, atrial fibrillation, uncontrolled pain, and MUA, with one patient requiring admission for atrial fibrillation and three for MUA. In the robotic group during the same period, complications included infection, cellulitis, deep vein thrombosis, low back pain, nausea, anaphylaxis, MUA, and atrial fibrillation, with patients requiring admission for revision due to infection, MUA, and atrial fibrillation.

**Fig. 2:**
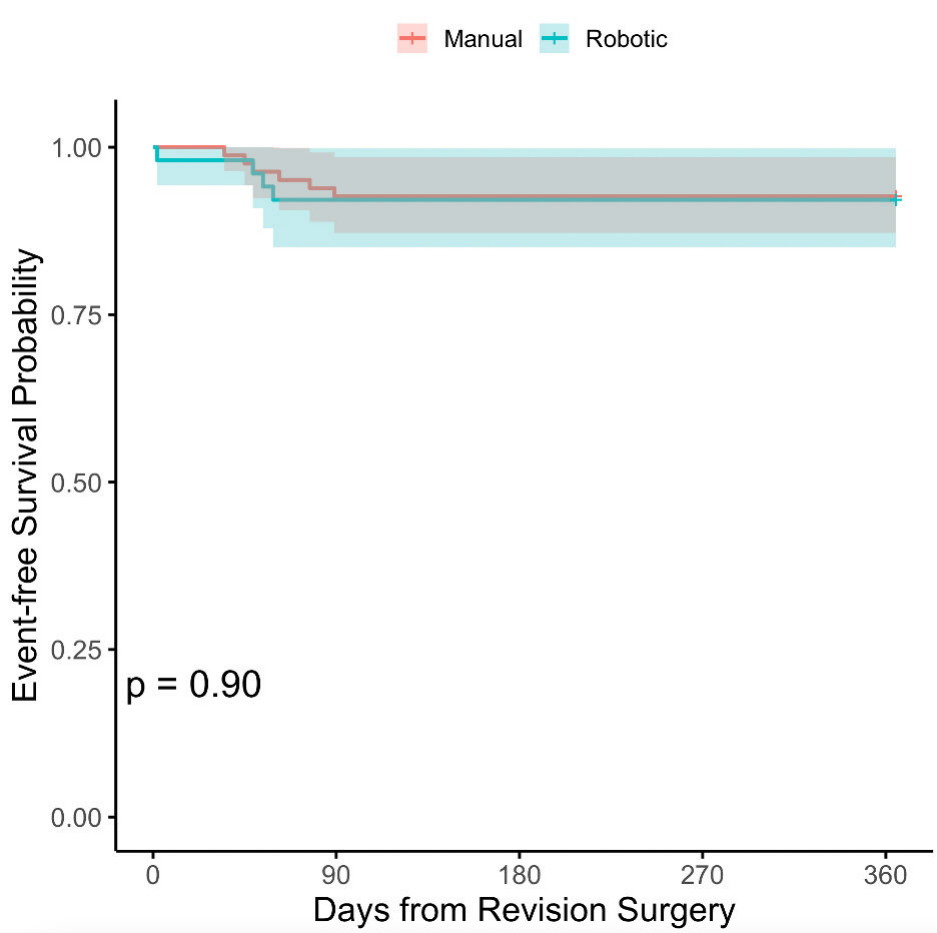
Kaplan-Meier curves demonstrating 365-day reoperation-free survival following revision total knee arthroplasty in patients undergoing manual (red) or robotic-assisted (blue) procedures. One-year reoperation-free survival was defined as the absence of manipulation under anaesthesia or rerevision. Shaded regions represent the 95% CIs. Log-rank test was used to compare survival distributions between groups. A p-value < 0.05 was considered statistically significant.

Two patients in the robotic group required MUA within one year, compared with six patients in the manual group (3.9% vs 7.3%; p = 0.710) (Table III). Each group had one patient who required a revision within one year, both due to infection (Table III). At one year postoperatively, reoperation-free survival was not significantly different between groups (91.5% vs 94.1%; p = 0.900; Figure 2). No significant differences in ROM were observed between groups at one year postoperatively (110° vs 114°) for the manual and robotic cohorts, respectively (p = 0.23) (Table III).

**Table III.** One-year clinical outcomes.

Outcome	Overall (n = 133)	Manual (n = 82)	Robotic (n = 51)	p-value*
Revision within 1 year, n (%)	2 (1.5)	1 (1.2)	1 (2.0)	1.000
MUA within 1 year, n (%)	8 (6.0)	6 (7.3)	2 (3.9)	0.710
Mean ROM at 1 year, ° (SD)	111 (12)	110 (12)	114 (12)	0.234

* One-year range of motion obtained at 12-month postoperative follow-up. Group differences assessed using Welch’s two-sample *t*-test. A p-value < 0.05 was considered statistically significant.

MUA, manipulation under anaesthesia; ROM, range of motion.

Mean KOOS-JR scores at three-month follow-up were similar between groups (60.6 vs 61.3; p = 0.445; Table II). Similarly, the change in KOOS-JR scores from preoperative baseline to three months did not differ between groups (p = 0.596).

## Discussion

Robotic-assisted primary TKA has been shown to improve implant alignment and gap symmetry, findings that in some studies have been associated with improved clinical outcomes; however, large registry analyses have not demonstrated a clear survivorship advantage compared with conventional manual techniques.^5,25^ Smaller cohort studies have described the successful implementation of robotic assistance in rTKA,^20,26^ yet evidence evaluating outcomes following rarTKA remains limited.^11,27^ To our knowledge, this study represents the first direct comparison of perioperative outcomes between manual and robotic-assisted rTKA performed using the MAKO robotic-arm system for revision due to knee instability.

Operating time was significantly longer in the rarTKA group compared with the manual rTKA group; however, the absolute difference was modest (nine minutes) and may not be clinically meaningful. This increase is partly attributable to the additional time required to obtain acceptable registration points, which can be distorted by metallic artefact from prior implants on preoperative CT imaging. Such limitations complicate achieving the 0.5 mm error threshold typically required for CT-based registration in revision procedures. Additionally, as rarTKA is an evolving technique, the lack of a standardized workflow contributes to prolonged operating times, particularly because certain procedural steps, such as preparation of stems and metaphyseal cones, are still performed with conventional instrumentation. Prior studies suggest that the learning curve associated with adopting new technology leads to a reduction in operating time as surgeon experience increases.^28^

Restoration of the joint line during rTKA is crucial for achieving optimal outcomes, as joint line position influences ROM, stability, and overall function.^29^ In this study, joint line reconstruction was achieved in both groups, with no statistically significant differences observed between the manual and robotic cohorts. Although joint line deviation was numerically smaller in the robotic cohort, potentially reflecting greater precision in bone preservation, this difference did not reach statistical significance. Notably, patients undergoing rarTKA demonstrated a significantly greater mean increase in PCO compared with the manual group, suggesting more accurate restoration of posterior femoral anatomy. This finding may be attributable to the enhanced precision of robotic-assisted planning and execution, including the ability to model sagittal plane balance throughout the ROM, which may facilitate improved flexion gap optimization.

A study evaluating pre- and postoperative component positioning in rarTKA reported average increases in posterior medial and lateral condylar offset of 4.2 mm and 3.3 mm, respectively, which aligns with the mean increase of 3.4 mm observed in the current study.^11^ This finding is particularly important given that all patients underwent revision for instability, suggesting that rarTKA may offer a meaningful advantage in restoring sagittal plane balance in this population. Prior studies by Clement et al^30^ and Ng et al^31^ have reported that PCO is an independent predictor of functional outcomes, with greater PCO correlating with higher patient satisfaction following rTKA. In the present study, however, the greater PCO restoration observed in the rarTKA cohort did not translate into differences in early patient-reported outcomes. This finding raises the possibility that the observed radiological difference may not be clinically meaningful in the short term. Larger studies with longer follow-up are required to determine whether improved PCO restoration confers sustained functional benefits over time.

Robotic revisions more commonly required both femoral (distal and posterior) and tibial augments, suggesting greater precision in defining and reconstructing the joint line and PCO. Additionally, while not statistically significant, the smaller average polyethylene thickness observed in the robotic group reinforces the notion of improved accuracy with the rarTKA technique. Robotic assistance enables precise 3D balancing during rTKA, as adjustments in one plane can produce subtle changes in other planes that influence gap balance throughout knee ROM. The reduced polyethylene thickness observed in the robotic cohort may therefore serve as a proxy for the enhanced precision afforded by this technique. This finding is consistent with prior studies on primary robotic TKA.^32^

Our study suggests that rarTKA may be associated with improved complication-free survival and reduced rates of MUA; however, these findings did not reach statistical significance. Early complication rates within 90 days were similar between the manual and robotic groups across a range of postoperative issues, reinforcing that early morbidity after rTKA is heterogeneous and likely not attributable solely to surgical technique. Importantly, rarTKA did not demonstrate any indication of increased risk for readmission, medical complications, or reoperation compared with manual rTKA, supporting its safety profile in the early postoperative period. Nonetheless, these findings should be interpreted with caution given the small sample size, which may limit the ability to detect significant differences.

One patient in each group required revision surgery for infection, and no additional rerevisions for instability or other causes were observed. Similarly, six patients in the manual group required postoperative MUA, compared with two patients in the robotic group. Although this finding did not reach statistical significance, it may suggest improved gap and soft-tissue balance with rarTKA; further research is warranted to validate this observation. One-year reoperation-free survival, defined as the absence of any rerevisions or MUA within 12 months, was comparable between the manual and robotic groups. Interpretation of this outcome, however, is limited by the modest sample size and the relatively short duration of follow-up. Prior literature suggests that a minimum follow-up of two years is often necessary to adequately capture early technical failures following rTKA, which may not have been fully represented in the present analysis.^33^ Additionally, patients undergoing rTKA are generally at higher risk for postoperative complications compared with those undergoing primary TKA,^34^ adding complexity to outcome interpretation in this population. Nevertheless, the absence of differences between techniques suggests that rarTKA may achieve an early safety profile comparable to that of manual revision for instability-related cases.

Although the robotic cohort used longer femoral stems on average, the manual group more frequently required offset couplers and diaphyseal-engaging stems. In contrast, offset couplers were not used in the rarTKA group, which may represent a potential cost consideration, although metaphyseal cones were used in both groups. The absence of offset couplers in the robotic cohort reflects a tendency towards shorter revision constructs and greater reliance on prepared residual bone stock for epiphyseal fixation rather than diaphyseal fixation.

Conventional rTKA techniques have historically emphasized diaphyseal fixation for stability, with reconstruction progressing toward the joint line. By contrast, rarTKA prioritizes establishment of the joint line as the initial reference point, from which the revision components are assembled, representing a potential departure from traditional revision philosophy. Notably, significantly fewer constrained polyethylene inserts were used in the robotic cohort compared with the manual cohort. Similarly, fewer femoral cones were required in the robotic group, further supporting the concept of improved bone preparation in conjunction with the use of augments for implant stability. Collectively, these findings align with a broader paradigm shift towards shorter revision constructs.^35,36^ However, the clinical implications of these construct differences, including their potential impact on implant survivorship or aseptic loosening, remain uncertain and were not evaluated in the present study.

There were no statistically significant differences in ROM between groups at two weeks or one year postoperatively, although the modest sample size may have limited the ability to detect a difference. Nevertheless, results from this retrospective study suggest that rarTKA may facilitate earlier ROM compared with manual rTKA, potentially due to improved knee balance achieved through greater symmetry between flexion and extension gaps and optimized soft-tissue tension. KOOS-JR scores at three months postoperatively demonstrated a similar trend. A larger patient cohort is needed to confirm or refute these observations.

Few studies have reported outcomes following rarTKA. The largest to date, describing the surgical technique and early outcomes using the Stryker MAKO robotic-arm system, include two studies by MacAskill et al^10,12^ and one by Danoff et al.^12^ However, neither of these studies directly compared manual TKA with rarTKA.

Cochrane et al^27^ reported their findings on rarTKA using the imageless CORI robotic platform (Smith & Nephew, USA). They evaluated all-cause revisions including instability, loosening, arthrofibrosis, and infection, whereas our study focused exclusively on revisions for instability. Their reported complication rate of 7% is comparable to the 7.7% observed in our cohort. Additionally, Cochrane et al^27^ noted an intervention rate, including manipulations and revision surgery, of 3% and a mean LOS of 2.3 days, which is similar to our cohort’s 5.8% reoperation rate but higher than the 1.3 day mean LOS observed in our study. A similar study using the CORI system demonstrated adequate joint line restoration and PCO maintenance, as well as improvements in pain scores postoperatively.^37^

This study has several limitations, including a small sample size limited to patients undergoing revision for mechanical complications, such as instability, and its conduct at a single centre by two fellowship-trained surgeons. These factors may introduce selection bias and limit generalizability of the findings. The retrospective design precluded random assignment to treatment groups, and all outcomes were limited to a maximum follow-up of one year, representing two major potential sources of bias. RarTKA is an emerging technique and is currently considered off-label for the robotic system used in this study. Therefore, these results should be interpreted as exploratory and hypothesis-generating rather than conclusive and should be evaluated with caution.

In conclusion, these findings indicate that rarTKA may achieve short-term outcomes comparable to those of manual rTKA, while demonstrating a significantly greater mean increase in PCO; however, the clinical significance of this radiological difference remains uncertain. Restoration of the joint line remains a critical objective in revision surgery, and improved accuracy in achieving this parameter may contribute to enhanced stability. Although operating times were longer in the robotic cohort, this difference likely reflects the learning curve associated with adoption of a novel technique. Given the retrospective design and limited sample size, these results should be interpreted as preliminary. Larger, prospective studies are needed to validate these findings and to determine whether robotic assistance confers meaningful clinical advantages in revision TKA.

**Take home message**

- Robotic-assisted revision total knee arthroplasty (TKA) for instability provided short-term clinical outcomes and complication rates comparable to conventional manual revision while achieving more accurate restoration of posterior condylar offset.

- These findings suggest that robotic assistance is a safe alternative for revision TKA, although longer-term follow-up is needed to determine whether improved radiological reconstruction translates into superior functional outcomes or implant survivorship.

## Data Availability

The datasets generated and analyzed in the current study are not publicly available due to data protection regulations. Access to data is limited to the researchers who have obtained permission for data processing. Further inquiries can be made to the corresponding author.
